# A Mill for the Toothless

**Published:** 1861-07

**Authors:** 


### Selections.

A Mill for the Toothless.—A little machine, intended for those who can not properly masticate their food, is advertised in the *Lancet.* It is fastened to the dinner-table, and minces the food very finely. We can imagine a table set with these labor-saving contrivances for the benefit of those who are toothless, or are too lazy to chew, and a party of grannies dining with motionless jaws, and simply turning a crank. The next inventive effort of the originator should be directed to an apparatus for mechanical *swallowing*, provided with a mould for the bolus, ramrod, etc.—*Medical and Surgical Reporter.*

